# Extended reality in skull base surgery: a systematic literature review

**DOI:** 10.3389/fsurg.2025.1642033

**Published:** 2025-12-15

**Authors:** Vivek Sanker, Emil O. R. Nordin, Amr Badary, Adam T. Eberle, Sudarshan Ramanan, Kate N. Jensen, Margaux E. Miller, Joshua S. Catapano, Anna L. Huguenard, Redi Rahmani, Michael T. Lawton

**Affiliations:** Department of Neurosurgery, Barrow Neurological Institute, St. Joseph’s Hospital and Medical Center, Phoenix, AZ, United States

**Keywords:** artificial intelligence, augmented reality, extended reality, machine learning, mixed reality, neurosurgery, otolaryngology, skull base

## Abstract

**Introduction:**

Extended reality (XR) technology may play an important role in progressing the field of skull base surgery. Its potential use in neurosurgical training, case preparation, and the operating room could make XR a powerful addition to the surgical toolbox. This study evaluated the application of XR in skull base surgery.

**Methods:**

A systematic literature search from inception to March 28, 2024, was performed using 4 databases: PubMed, Scopus, Web of Science Advance, and Embase (Ovid). Original studies involving the use of XR in skull base surgery for surgical planning or training purposes were included. Conference abstracts, reviews, and case reports were excluded.

**Results:**

Of 357 articles screened across all 4 databases, 250 were included. After careful evaluation of titles and abstracts for eligibility, 29 articles were deemed suitable for full-text examination. A subsequent detailed assessment excluded 8 articles, resulting in a final 21 studies that met the criteria for inclusion in the systematic review. Of the 21 studies included, 13 (62%) focused on augmented reality, 4 (19%) focused on virtual reality, and 4 (19%) focused on mixed reality. Augmented reality has demonstrated varying degrees of effectiveness, with mean registration accuracy reported between 2.5 and 10.75 mm. The mean (SD) registration error reported in mixed reality was 5.76 (0.54) mm. Virtual reality has been used for preoperative planning and intraoperative guidance, with average computation times ranging from 15 s to 2 min.

**Discussion:**

The role of XR in skull base surgery is anticipated to grow, given its potential for streamlining surgical planning, neuronavigation, and teaching. Although the use of XR in skull base surgery shows promise, the technologies associated with these modalities require substantial improvement before XR is a stable component of the neurosurgical toolbox.

## 1 Introduction

In 1935, science fiction writer Stanley Weinbaum presented readers with a fascinating new premise in his novel *Pygmalion's Spectacles*: What is it like to explore a new world using a pair of goggles? (1) Although appearing in a fictional story, this idea planted the seed for the conception of extended reality (XR), or any form of technology that uses digital elements to alter perception of a real-world environment (2). There are 3 primary forms of XR. Augmented reality (AR) involves digital elements that are layered over a physical environment to create a composite image (3). Mixed reality (MR) is an extension of AR in which users can interact with digital elements that are overlaid on a physical environment (3). Lastly, virtual reality (VR) enables individuals to immerse themselves in a fully digital environment and interact with it (4). In recent years, XR has been introduced to a variety of industries, including entertainment, manufacturing, and healthcare, primarily due to breakthroughs in software and graphic design, as well as significant hardware improvements (5–7). Given the current applications and unique potential of XR in these industries and sectors, it is unsurprising that XR has also begun to impact the field of skull base surgery.

Skull base surgery is a highly specialized field of neurosurgery and otolaryngology (8). Due to the presence of vital and eloquent structures in the head, face, and neck, the skull base has traditionally been one of the most difficult areas to access surgically (9, 10). Skull base approaches are segregated into 2 categories: open and endoscopic (11). Traditional open skull base approaches involve a more extensive craniotomy, and endoscopic surgery offers alternative approaches that are typically associated with lower morbidity and complication rates (12–14).

Because of their complexity, skull base procedures require an immense degree of precision and technical expertise. Any advancements in XR technologies that can provide surgeons with the most up-to-date training and guidance are welcomed. The use of XR in skull base surgery can be segmented into 2 roles: training, and perioperative and intraoperative aid. This study reviewed the use of XR technology in each of these roles and evaluated the future implications of XR in skull base surgery.

## 2 Methods

### 2.1 Search strategy

The PubMed, Scopus, Web of Science Advance, and Embase (Ovid) databases were retrospectively queried to identify all relevant studies from inception to March 28, 2024, using a search string with the following keywords: “extended reality,” “augmented reality,” “virtual reality,” “mixed reality,” and “skull base surgery” (Table 1). Individuals of all ages were considered for inclusion. Studies that were either unrelated to XR or did not include patients undergoing skull base procedures were excluded. Animal studies, reviews, and nonoriginal research articles were also excluded from analysis.

**Table 1:** Adjusted search terms for electronic databases searched on March 28, 2024.

Database	Search query	Results
Embase	(“extended reality”:ti OR “augmented reality”:ti OR “virtual reality”:ti OR “mixed reality”:ti) AND “skull base surgery”:ti	15
PubMed	((“skull base surg”[Journal] OR (“skull”[All Fields] AND “base”[All Fields] AND “surgery”[All Fields]) OR “skull base surgery”[All Fields]) AND (((“extend”[All Fields] OR “extendable”[All Fields] OR “extended”[All Fields] OR “extendibility”[All Fields] OR “extendible”[All Fields] OR “extending”[All Fields] OR “extends”[All Fields]) AND (“realities”[All Fields] OR “reality”[All Fields])) OR (“virtual reality”[MeSH Terms] OR (“virtual”[All Fields] AND “reality”[All Fields]) OR “virtual reality”[All Fields]) OR (“augmented reality”[MeSH Terms] OR (“augmented”[All Fields] AND “reality”[All Fields]) OR “augmented reality”[All Fields]) OR (“augmented reality”[MeSH Terms] OR (“augmented”[All Fields] AND “reality”[All Fields]) OR “augmented reality”[All Fields] OR (“mixed”[All Fields] AND “reality”[All Fields]) OR “mixed reality”[All Fields]))) AND ((ffrft[Filter]) AND (english[Filter]))	40
Scopus	(TITLE-ABS-KEY (extended AND reality) OR TITLE-ABS-KEY (augmented AND reality) OR TITLE-ABS-KEY (virtual AND reality) OR TITLE-ABS-KEY (mixed AND reality) AND TITLE-ABS-KEY (skull AND base AND surgery)) AND (LIMIT-TO (LANGUAGE, “English”)) AND (LIMIT-TO (DOCTYPE, “ar”))	124
Web of Science	((((ALL = (Extended reality)) OR ALL = (Mixed reality)) OR ALL = (Virtual reality)) OR ALL = (Augmented reality)) AND ALL = (Skull base surgery) and Article (Document Types) and English (Languages)	178

### 2.2 Screening of studies

**Figure 1:**
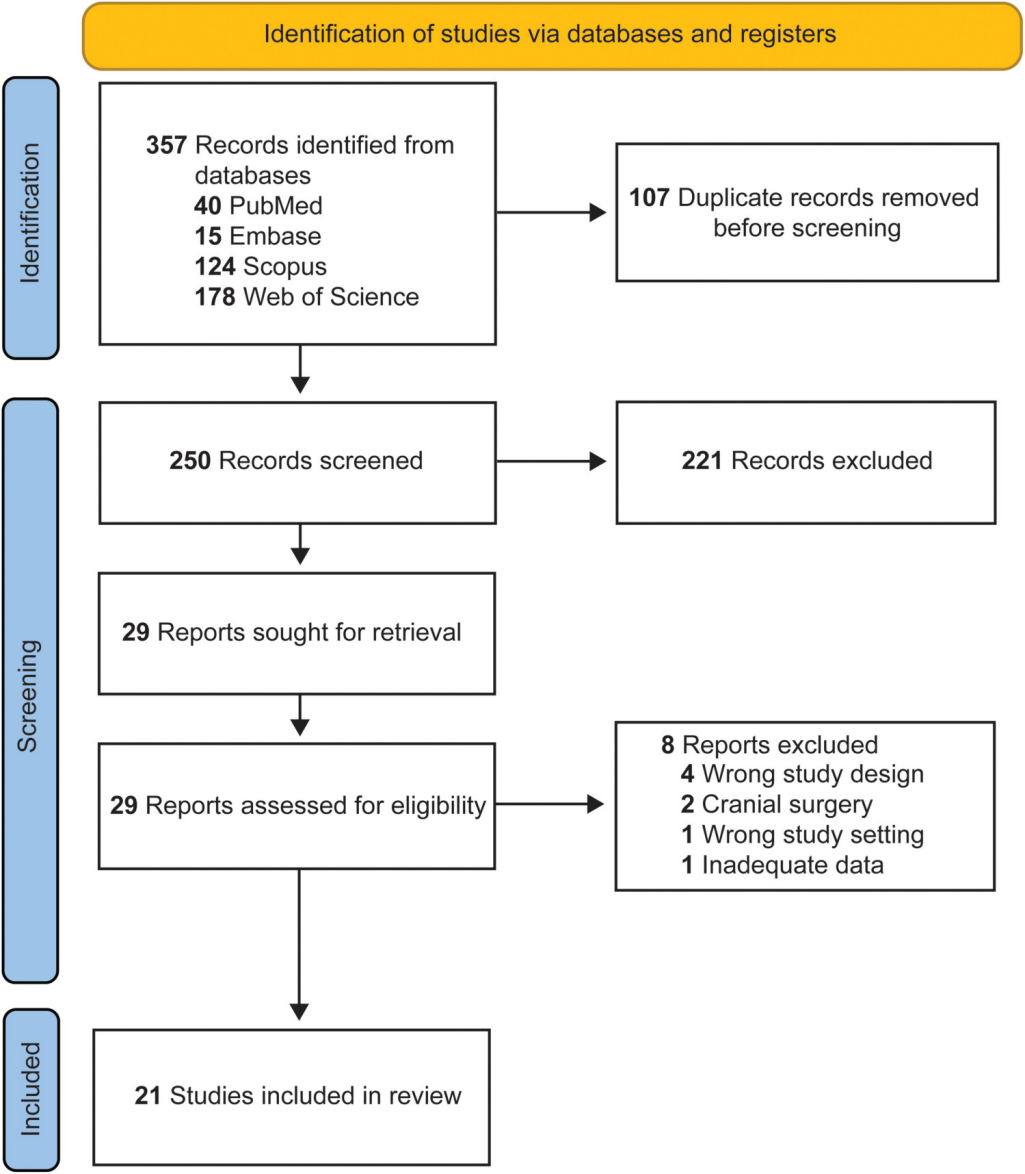
Preferred Reporting Items for Systematic Reviews and Meta-Analyses flow diagram summarizing the article screening process*.* Used with permission from Barrow Neurological Institute, Phoenix, Arizona.

The initial title and abstract screening was led by 2 authors (V.S. and E.N.). Any discrepancies were addressed through consultation with a third co-author (A.B.) and further discussion with all authors. The screening of studies adhered to Preferred Reporting Items for Systematic Reviews and Meta Analyses guidelines (Figure 1).

### 2.3 Data extraction

Three independent authors (V.S., E.N., and A.B.) extracted relevant data from the selected studies. The data collected included study design, participant demographics, and the number of participants with respective outcomes and complications. Discrepancies in data extraction were resolved through consensus, and any unresolved disagreements were addressed through consultation with a fourth reviewer (A.T.E.). The results are summarized and discussed on the basis of the salient features extracted from the studies in the AR, MR, and VR categories. We included ventriculostomy and external ventricular drain simulations under “skull base surgery” due to their anatomical proximity to the skull base and their relevance in training to manage complications associated with skull base interventions. However, due to the limited data and insufficient published research, we were unable to determine the statistical significance among the various XR technologies and performance outcomes in skull base neurosurgery.

## 3 Results

### 3.1 Study selection

A total of 357 articles underwent initial screening, after which 107 duplicate articles were identified and subsequently removed. Careful evaluation of titles and abstracts yielded 29 articles that were deemed suitable for full-text examination. A detailed assessment excluded 8 articles; thus, 21 studies met the inclusion criteria and were incorporated into our study (Tables 2–4) (15–35).

**Table 2:** Summary of augmented reality (AR) studies.

Study	Title	Publication year	Study setting	Procedure	Diagnosis	No. of Patients	Role of AR
Goto et al. (18)	Efficacy of a Novel Augmented Reality System Using 3D Computer Graphic Modeling in Endoscopic Transsphenoidal Surgery for Sellar and Parasellar Tumors	2023	Human study	Endoscopic transsphenoidal surgery	Tumors	15	Navigation and visualization
El Chemaly et al. (16)	Stereoscopic Calibration for Augmented Reality Visualization in Microscopic Surgery	2023	Phantom study	Middle and inner ear procedures	Model	Model	Navigation and visualization
Ansari et al. (15)	VentroAR: An Augmented Reality Platform for Ventriculostomy Using the Microsoft HoloLens	2023	Phantom study	Ventriculostomy	Model	Model	Navigation and visualization
Steiert et al. (21)	Augmented Reality-Assisted Craniofacial Reconstruction in Skull Base Lesions–An Innovative Technique for Single-Step Resection and Cranioplasty in Neurosurgery	2022	Phantom study	Cranioplasty	Model	Model	Navigation and visualization
Pojskic et al. (20)	Microscope-Based Augmented Reality with Intraoperative Computed Tomography-Based Navigation for Resection of Skull Base Meningiomas in Consecutive Series of 39 Patients	2022	Human study	Craniotomy	Meningioma	39	Navigation and visualization
Leuze et al. (19)	Augmented Reality for Retrosigmoid Craniotomy Planning	2022	Cadaver study	Craniotomy	Tumors	14	Surgical planning
Gibby et al. (17)	The Application of Augmented Reality-Based Navigation for Accurate Target Acquisition of Deep Brain Sites: Advances in Neurosurgical Guidance	2022	Phantom study	Biopsy and other minimally invasive neurosurgical procedures	Model	Model	Navigation
Lai et al. (22)	Fusion of Augmented Reality Imaging with the Endoscopic View for Endonasal Skull Base Surgery; A Novel Application for Surgical Navigation Based on Intraoperative Cone Beam Computed Tomography and Optical Tracking	2020	Phantom study	Endoscopic endonasal surgery	Model	Model	Navigation and visualization
Creighton et al. (24)	Early Feasibility Studies of Augmented Reality Navigation for Lateral Skull Base Surgery	2020	Phantom study	Anatomical surface mapping	Model	Model	Navigation
Bong et al. (23)	Endoscopic Navigation System With Extended Field of View Using Augmented Reality Technology	2018	Phantom study	Endoscopic skull base surgery	Model	Model	Navigation and visualization
Li et al. (28)	A Novel Augmented Reality Navigation System for Endoscopic Sinus and Skull Base Surgery: A Feasibility Study	2016	Cadaver and phantom study	Endoscopic sinus and skull base surgery	None and model	15 and model	Navigation and visualization
Paul et al. (32)	Augmented Reality Based on Stereoscopic Reconstruction in Multimodal Image-Guided Neurosurgery: Methods and Performance Evaluation	2005	Human study	Craniotomy	3 Cavernomas; 3 low-grade tumors	6	Navigation and visualization
Freysinger et al. (27)	Image-Guided Endoscopic ENT Surgery	1997	Human study	Endoscopic surgery	Sinusitis, biopsy, orbital decompression, tumor, foreign bodies	79	Navigation

3D, 3-dimensional; ENT, ear, nose, and throat.

**Table 3:** Summary of virtual reality (VR) studies.

Study	Title	Publication year	Study setting	Procedure	Diagnosis	No. of patients	Role of VR
Filimonov et al. (26)	Virtual Reality Surgical Planning for Endoscopic Endonasal Approaches to the Craniovertebral Junction	2022	Human study	Endoscopic endonasal surgery	Various pathologies of the craniovertebral junction	5	Surgical planning
Liu and Yi (29)	A New Bony Anatomical Landmark for Lateral Skull Base Surgery	2020	Cadaver study	Craniotomy	None	15	Mapping of new anatomical landmark
Won et al. (34)	Early Experience with a Patient-Specific Virtual Surgical Simulation for Rehearsal of Endoscopic Skull-Base Surgery	2018	Human study	Endoscopic skull base surgery	Various sinus and ventral skull base pathologies	10	Surgical planning
Rosahl et al. (33)	Virtual Reality Augmentation in Skull Base Surgery	2006	Human study	Craniotomy	Tumors of the anterior, middle, and posterior skull base	110	Surgical planning

**Table 4:** Summary of mixed reality (MR) studies.

Study	Title	Year	Study Setting	Procedure	Diagnosis	No. of Patients	Role of MR
Marrone et al. (30)	Improving Mixed-Reality Neuronavigation with Blue-Green Light: A Comparative Multimodal Laboratory Study	2024	Phantom study	Locating specific markers on the plaster surface	Model	29	Navigation
Eom et al. (25)	Accuracy of Routine External Ventricular Drain Placement Following Mixed Reality-Guided Twist-Drill Craniostomy	2023	Phantom study	Craniostomy	Model	Model	Navigation
Zeiger et al. (35)	Use of Mixed Reality Visualization in Endoscopic Endonasal Skull Base Surgery	2020	Human study	Endoscopic endonasal skull base surgery	Various skull base pathologies	134	Navigation and visualization
McJunkin et al. (31)	Development of a Mixed Reality Platform for Lateral Skull Base Anatomy	2018	Phantom study	Locating anatomical landmarks	Model	Model	Navigation and visualization

### 3.2 Study characteristics

Of the 21 studies included, 13 focused on AR, and 4 each focused on VR and MR. The detailed characteristics of each study are presented in Tables 2–4 (15–35).

### 3.3 AR in skull base surgery

Blending AR with real-world intraoperative imaging and navigation systems allows visualization of critical neuroanatomical structures in real time and can aid in the resection of skull base pathologies (36). For example, in a report from 2018, Umebayashi et al. described employing AR-enhanced navigation maps to guide electric drills during procedures (37). Similarly, in 2020, Jean demonstrated the use of 3-dimensional (3D) VR renderings for preoperative rehearsal and intraoperative guidance in a minipterional craniotomy approach, highlighting AR's capacity to improve surgical accuracy and outcomes by displaying the anticipated surgical opening as an overlay over the patient in real time (38).

In 2023, Goto et al. evaluated a novel AR navigation system for endoscopic transsphenoidal surgery, and its accuracy was measured in 15 consecutive patients using a 5-point scale, on which a 4 represented “as useful as conventional neuronavigation,” and a 5 represented “more useful than conventional neuronavigation.” In their study, the AR navigation received a mean score of 4.7 (95% CI: 4.58–4.82), indicating that AR navigation was more useful than conventional navigation in most cases. However, depth perception of the lesion was more difficult with AR navigation in 2 cases (a skull base chondrosarcoma and craniopharyngioma) (18). In 2023, El Chemaly et al. assessed stereoscopic calibration for AR in microsurgery, in which the intrinsic (e.g., principal point, focal length, lens distortion) and extrinsic (location and orientation) parameters of the surgical stereo microscope were calculated using a calibration board that was 3D printed and electromagnetically tracked. The electromagnetic tracker coordinate system and the stereo microscope image space were transformed using these parameters such that any tracked 3D point could be projected onto the left and right visuals of the microscope video stream. Calibration was done once the system was set up, and an average calibration workflow time of 55.5 s and an average computational time of 10.5 s were reported, both being critical components of operational efficiency (16).

The microscope can be efficiently precalibrated at multiple focal lengths and the corresponding intrinsic parameters can be obtained. Zooming or changing the focal length of the microscope necessitates recalibration; thus, algorithms that provide automatic camera parameter calibration from a single image taken in any position increase efficiency. In clinical settings, this alternate technique allows for rapid recalibration if the focal length is changed, decreasing the total time required to adjust the surgical equipment.

In 2023, Ansari et al. introduced the VentroAR platform for ventriculostomy, with metrics showing a mean (SD) targeting accuracy of 10.64 (5.0) mm, which was measured in terms of the distance of the user's hitting point to the target or the total error. The mean (SD) targeting accuracy of the free hand group after training was 16.7 (5.4) mm in a 2021 study by Van Gestel et al. (39). In a 2022 study by Steiert et al., AR-assisted cranioplasty was employed for single-step resection and cranioplasty, although detailed quantitative results were sparse (21). In a 2022 study, Pojskic et al. combined AR with intraoperative computed tomography navigation for meningioma resections, showing promising outcomes in terms of neuronavigation accuracy and resection success (20). Gross total resection was achieved in 26 of 39 (66.7%) patients, a rate the authors describe as consistent with the published data for similar skull base meningiomas. The accuracy of AR systems is often quantified using target registration error (TRE), which measures the alignment between projected and actual anatomical structures. Automatic registration applying intraoperative computed tomography resulted in high accuracy in registering a target point to a model, which had a mean (SD) TRE of 0.82 (0.37) mm. This measurement is dramatically lower than landmark-based neuronavigation, which had a mean (SD) TRE of 1.85 (1.02) mm, as shown in the study by Bopp et al. (40). Additional AR studies described various applications and outcomes, including a study on retrosigmoid craniotomy planning and AR integration with endoscopic views, underscoring AR's diverse applicability in enhancing surgical precision and workflow (19, 22).

AR technologies have demonstrated varying degrees of accuracy depending on the specific applications, with studies reporting TRE as low as 0.82 mm with the use of intraoperative computed tomography. Reported calibration times in AR studies range from 0.11 to 0.94 s. Although to date few studies have reported improved outcomes associated with AR-assisted surgery, it is important to note that surgical outcomes are not consistently reported across all studies. Among the studies in which outcomes were detailed, there were 60 gross total resections (18, 20, 21), 3 near-total resections (18), 15 subtotal resections (18, 20, 21), 10 partial resections (20, 21), and 5 biopsies [including 2 open biopsies (20, 21) and 3 intraoperative biopsies (21)]. Although specific complication rates are reported far less frequently, the general efficacy of AR in improving surgical navigation and outcomes is well-documented. For example, AR-assisted cranioplasty and endoscopic transsphenoidal surgery show promising results in enhancing accuracy and reducing procedural errors (18).

However, this advancement in AR-guided neuronavigation comes with an added financial burden to the healthcare facility. A study by Davis et al. showed that the total cost of setting up Virtual Interactive Presence in Augmented Reality for a year would be $14,930.39 (41). The Vuzix smart spectacles and the Proximie system retail for $17,000 and $1,300–$2,500 annually, respectively (42). AR software licenses cost $7,000, and hardware costs $2,200, based on the study by Vyas et al. (43). Even though these rates are specific for each region, the cost of a number of these characteristics differs based on the location and the limited supply of goods and services in the area.

### 3.3 Role of AR as an educational tool in skull base surgery

#### 3.3.1 Ventriculostomy

Several studies have investigated the utility of AR in the training of ventriculostomy (39, 44, 45). In one study, 3D-reconstructed ventricles were superimposed on a phantom head using a smartphone display, whereas in 2 other experiments, a head-mounted display was used. One study directly contrasted AR training with conventional training techniques (39). In comparison with the freehand group [mean (SD) TRE: 19.9 (4.2) mm, *p* = 0.003], the AR group's external ventricular drain placement was noticeably more accurate [mean (SD) TRE for the untrained group: 11.9 (4.5) mm] (39). Additionally, those who underwent AR training had considerably better insertion performances (59.4% modified Kakarla scale grade 1) than those who received freehand training (25.0% modified Kakarla scale grade 1, *p* = 0.005) (39). Schneider et al. detailed a comparable AR-based external ventricular drain installation training simulator in a different study (44). 3D ventricles were registered using a head-mounted display and QR codes affixed to a model skull. Although freehand insertion was not used as a control group in this investigation, the authors observed a mean (SD) TRE of 2.71 (1.18) mm and a ventricle hit rate of 68.2% when using AR (44).

#### 3.3.2 Neuroanatomy teaching

A small number of studies examined how well AR interventions worked in comparison with more conventional approaches for teaching neuroanatomy, but the majority were not created with skull base surgical training in mind (46–50). Written tests were used to evaluate knowledge results in 4 out of 5 trials. Only 1 study found that the AR intervention increased understanding of neuroanatomy (46). One study revealed that traditional 2-dimensional learning approaches yielded more learning gain than AR-based methods, whereas 2 studies found no effect of AR training on examination scores (47, 49). Post-intervention surveys and satisfaction ratings showed that participants preferred learning neuroanatomy with AR-based approaches, despite the equivocal effect of AR on neuroanatomy examination performances.

### 3.4 MR in skull base surgery

A 2024 study by Marrone et al. compared standard magnetic neuronavigation (Medtronic Stealth Station S8) with MR neuronavigation under different lighting conditions (30). Although detailed quantitative results were not provided, MR navigation was noted to enhance visualization and potentially improve surgical accuracy. In another study, Eom et al. investigated MR-guided external ventricular drain placement, in which user trials consisted of 1 blind and 1 MR-assisted drilling of a burr hole followed by a routine, unguided external ventricular drain catheter placement for each of 2 different drill bit sizes. The mean (SD) distance from the catheter target improved from 18.6 (12.5) mm to 12.7 (11.3) mm (*p* < 0.001) using MR guidance for trials with a large drill bit and from 19.3 (12.7) mm to 10.1 (8.4) mm with a small drill bit (*p* < 0.001) (25). This study found that real-time quantitative and visual feedback of an MR-guided burr hole procedure can independently improve procedural accuracy. Zeiger et al. reported the utility of MR in various endoscopic skull base procedures, including pituitary tumors and anterior skull base meningiomas, in a cohort of 134 patients (35). Surgeons in this series reported that the technology was particularly useful in less commonly used surgical approaches, such as transorbital surgery. The series reported no intraoperative complications, although some patients did report postoperative complications following surgery of the anterior skull base (35). A 2018 study by McJunkin et al. focused on the use of MR for lateral skull base anatomy, emphasizing the potential of MR to enhance anatomical understanding and precision in temporal bone surgery (31).

The mean (SD) registration error reported in all included MR studies is 5.76 (0.54) mm (35). It is usually measured as the average distance between the virtual object placed in the real world and its intended position, often using specific markers or reference points. In a series of 118 tumor surgeries using MR, reported outcomes included a postoperative death due to respiratory failure (1.5%), hospital readmission (6.7%), and other issues such as cerebrospinal fluid leaks and visual defects, not necessarily due to the use of MR itself. In these cases, resection outcomes varied, with gross total resection achieved in approximately 56.3% of cases and subtotal resection achieved in 43.7% of cases (35). Despite some variability in procedural details and accuracy metrics, MR demonstrates promising potential for enhancing surgical precision and improving patient outcomes.

### 3.5 Role of MR as an educational tool in skull base surgery

#### 3.5.1 Brain tumor surgery

The viability of using an MR device for neurosurgery education was investigated by Jain et al. using 3 case scenarios, including that of a sphenoid wing meningioma (51). The study also assessed the trainees' experiences with the MR platform. The study involved the recruitment of 8 neurosurgeon trainees. For most trainees, the learning curve was low even though they had never used an MR platform before. The trainees' answers to the question of whether MR could replace the conventional approaches currently used in teaching neuroanatomy were split. The trainees rated the device as appealing, dependable, innovative, and easy to use, as shown by the user experience questionnaire results.

#### 3.5.2 Neurosurgery teaching

In a quasi-experimental study of 223 medical students (120 in the conventional group and 103 in the MR group), Isidre et al. sought to determine whether an MR-guided neurosurgical simulation module in the context of an undergraduate neurosurgical hands-on course could increase medical student satisfaction (52). The mean (SD) satisfaction scores for the conventional group [89.3 (13.3)] and MR group [94.2 (7.5)] indicated that using MR-simulation was linked to higher levels of satisfaction.

### 3.6 VR in skull base surgery

VR technology has been used for preoperative planning and intraoperative guidance in skull base surgery. A study by Filimonov et al. explored VR applications for endoscopic transnasal approaches to the craniovertebral junction, noting that VR systems provided valuable preoperative insights, including safe surgical planning and visualization of critical structures, despite the small sample size (*n* = 5) (26). A 2020 study by Liu and Yi identified a new bony landmark (point O, which is the junction point of the temporosphenoid suture and the infratemporal ridge) for lateral skull base surgery using VR, focusing on anatomical precision in cadaveric studies, and the distances measured were in agreement with the actual distances obtained by cadaveric dissections. The mean (SD) dissection vs. mean (SD) VR 3D stereoscopic image was 22.52 (2.47) vs. 22.42 (2.85) for point O to the foramen rotundum (*p* = 0.81), 22.62 (2.60) vs. 23.51 (2.09) for point O to the foramen spinosum (*p* = 0.67), 23.69 (2.34) vs. 22.77 (2.90) for point O to the foramen ovale (*p* = 0.70), and 24.42 (2.38) vs. 24.19 (2.65) for point O to the superior orbital fissure (*p* = 0.59) (29). The early feasibility study by Won et al. highlighted VR's potential in rehearsing complex endoscopic skull base procedures, with a reported simulation time between 1 and 2 h depending on the number and complexity of the segmented structures, concurrently allowing the surgeon to begin a virtual dissection of the sinuses and ventral skull base (34). In a 2006 study, Rosahl et al. demonstrated the application of VR in enhancing the surgical field through image guidance, although detailed statistics on the impact of VR on surgical outcomes were not extensively documented (33).

The average computation times for VR simulations ranged from 15 s to 2 min, reflecting variability based on the surgeon's experience in this emerging modality. Complications reported in these studies were not due to the VR systems themselves but included intraoperative cerebrospinal leaks and prolonged intubation, although detailed rates were not uniformly provided. Although VR studies do not consistently report specific accuracy metrics, the role of VR in enhancing surgical planning and visualization is evident.

### 3.7 Role of VR as an educational tool in skull base surgery

In training environments, high-fidelity VR systems enable surgeons to practice procedures on virtual patients, providing scalable and cost-effective alternatives to traditional cadaveric models (53). These fully digitized surgeries also introduce new methods for assessing surgical skills by capturing performance metrics that are challenging to measure in real-world scenarios (54).

#### 3.7.1 Neuroanatomy education

A 2014 study by Arora et al. examined the viability of performing case-specific surgical rehearsal using a VR temporal bone simulator (55). They conducted 3 dissection tasks on the case simulation and cadaver models after completing a 90-min temporal bone dissection on the general simulation model. The usefulness of VR as an educational tool is demonstrated by the high ratings (Likert score >4) given to case rehearsal for confidence (75%), training (94%), and planning facilitation (75%).

Another study consisting of 16 otolaryngology and head-and-neck surgical residents reported a significant increase in overall confidence in 87.5% of the participants after conducting an anatomy-specific VR rehearsal (56). Similar studies have demonstrated the role of VR simulation as an effective training aid for temporal bone surgery (57–60). In a pilot study, Munawar et al. reported on the use of the Fully Immersive Virtual Reality System for skull-base surgery, which combines high-fidelity hardware with surgical simulation software, to perform virtual cortical mastoidectomy (54). There were 7 participants, including 3 attending surgeons, 3 residents, and a medical student. The preliminary data collected by this system distinguished between participants with varying levels of ability, showing a great promise for automatic skill assessment.

#### 3.7.2 Skull base tumor surgery

In a study by Shao et al., 30 undergraduate students were randomly divided into a virtual reality teaching group and a traditional teaching group for a set of 10 cases of skull base tumors (61). A comparison of the 2 groups showed that the virtual reality teaching group had a better response effect than the traditional teaching group. Response effect was a knowledge assessment comparing the 2 groups. The VR group outperformed the traditional instruction group in terms of basic theory, location, adjacent structure, clinical manifestation, diagnosis and analysis, surgical procedures, and overall scores.

### 3.8 XR in skull base surgery

The comparative analysis of XR, based on the limited literature available, found that the pooled mean registration error was 5.76 mm for MR, 6.0 mm for VR, and 6.5 mm for AR (31). Integrating MR, VR, and AR technologies into skull base surgery aims to significantly enhance surgical precision and planning, with no evidence suggestive of increased complications. Overall, MR technology was found to offer improved registration accuracy and resection outcomes, VR technology was found to enhance preoperative planning and simulation, and AR technology was found to provide real-time neuronavigation improvements. The role of XR as an educational tool in skull base surgery is summarized in Table 5 (39, 44, 46–52, 54–56, 61).

**Table 5:** Summary of XR studies as educational tool.

Augmented reality			
Study	Title	Objective	Key Findings
Ventriculostomy			
Van Gestel et al. (39)	The effect of augmented reality on the accuracy and learning curve of external ventricular drain placement	To demonstrate the impact of AR-assistance on the accuracy and learning curve of EVD placement compared with the freehand technique	Untrained (11.9 ± 4.5 mm) and trained (12.2 ± 4.7 mm) AR performances were significantly better than the untrained freehand performance (19.9 ± 4.2 mm), which improved after training (13.5 ± 4.7 mm). Both untrained and trained AR performances (59.4% mKS grade 1 for both) were significantly better than the untrained freehand performance (25.0% mKS grade 1).
Schneider et al. (44)	Augmented reality–assisted ventriculostomy	To develop a compact navigational AR–based tool that does not require rigid patient head fixation, to support the surgeon during the operation	Findings showed an overall ventriculostomy success rate of 68.2%. The mean offset from the reference trajectory as displayed in the hologram was 5.2 ± 2.6 mm and a mean (SD) target error of 2.71 ± 1.18 mm.
Neuroanatomy Teaching			
Fernandes et al. (46)	An augmented reality-based mobile application facilitates the learning about the spinal cord	To investigate a mobile application with AR technology named *NitLabEduca* for studying the spinal cord with an interactive exploration of 3D rotating models	Studying the spinal cord anatomy through *NitLabEduca* seems to favor learning when used as a complement to the printed material.
Henssen et al. (47)	Neuroanatomy learning: augmented reality vs. cross-sections	To investigate the differences on test scores, cognitive load, and motivation after neuroanatomy learning using AR applications and using cross-sections of the brain	Students who worked with cross-sections (*n* = 16) showed significantly more improvement on test scores than students who worked with *GreyMapp-AR* (*P* = 0.035) (*n* = 15).
Ille et al. (48)	Augmented reality for the virtual dissection of white matter pathways	To test and evaluate a new method for fiber dissection using AR in a group experienced in cadaver white matter dissection courses and *in vivo* tractography	Participants rated the overall experience of AR fiber dissection with a median of 8 points (mean ± standard deviation 8.5 ± 1.4). Usefulness for fiber dissection courses and education in general was rated with 8 (8.3 ± 1.4) and 8 (8.1 ± 1.5) points, respectively.
Mendez-Lopez et al. (49)	Evaluation of an augmented reality application for learning neuroanatomy in psychology	To compare neuroanatomy learning activity using one of the visualization methods: a 3D method using a mobile AR application and a two-dimensional method using a textbook to color, followed by questions concerning their satisfaction and knowledge	Results showed that the AR application was highly valued by students and was as effective as the textbook.
Mixed reality			
Study	Title	Objective	Key Findings
Brain tumor surgery			
Jain et al. (51)	Use of mixed reality in neurosurgery training: a single centre experience	To determine the feasibility of employing an MR device in a high-volume center for neurosurgical teaching	The study showed the feasibility of using an MR platform such as HoloLens 2 (Microsoft) in neurosurgery training without significant preparation requirements, with positive trainee feedback.
Neurosurgical teaching			
Pickering et al. (50)	Assessing the difference in learning gain between a mixed reality application and drawing screencasts in neuroanatomy	To assess the impact of a mixed reality application in comparison with an existing resource already embedded within curriculum (anatomy drawing screencasts), focused on long spinal cord sensory and motor pathways	Screencast group showed a stronger impact on information retention than MR application groups, though both the groups showed a significant improvement in knowledge retention.
Isidre et al. (52)	Mixed reality as a teaching tool for medical students in neurosurgery	To identify whether an MR neurosurgical simulation module within the setting of an undergraduate neurosurgical hands-on course could improve the satisfaction of medical students	The satisfaction scores for the control group and mixed reality group were 89.3 ± 13.3 and 94.2 ± 7.5, respectively. This study reports a positive response toward MR as an educational tool.

Virtual reality			
Study	Title	Objective	Key Findings
Brain tumor surgery			
Shao et al. (61)	Virtual reality technology for teaching neurosurgery of skull base tumor	To assess the teaching effectiveness between two groups: the traditional teaching group and the VR teaching group, based on basic theoretical knowledge, case analysis, and questionnaire survey methods	The response effect of the VR teaching group was better than that of the traditional teaching group (*P* < 0.05). Also, the scores of basic theory, location, adjacent structure, clinical manifestation, diagnosis and analysis, surgical methods, and total scores in the VR group exceeded those in the traditional teaching group (*P* < 0.05).
Neuroanatomy teaching			
Arora et al. (55)	Virtual reality case-specific rehearsal in temporal bone surgery: a preliminary evaluation	To investigate the feasibility of performing case-specific surgical rehearsal using a VR temporal bone simulator	Case rehearsal rated highly (Likert score >4) for confidence (75%), facilitating planning (75%), and training (94%), showing the utility of VR in surgical rehearsal.
Locketz et al. (56)	Anatomy-specific virtual reality simulation in temporal bone dissection: perceived utility and impact on surgeon confidence	To evaluate the effect of anatomy-specific VR surgical rehearsal on surgeon confidence and temporal bone dissection performance	Fourteen subjects reported a significant increase in overall confidence after conducting an anatomy-specific VR rehearsal.
Munawar et al. (54)	Fully immersive virtual reality for skull-base surgery: surgical training and beyond	To present a fully immersive virtual reality system for skull base surgery, which combines surgical simulation software with a high-fidelity hardware setup	The study is primarily limited to showcase the utility of the system as a simulation system combining software and hardware components for immersive training, which also records data for quantitative skill assessment.

AR, augmented reality; CT, computed tomography; EVD, external ventricular drain; mKS, modified Kakarla scale; MR, mixed reality; VR, virtual reality; XR, extended reality.

## 4 Discussion

This systematic review included 21 articles and aims to provide an overview of the current research on XR utilization in skull base surgery. To facilitate improved digital representations for training and surgical planning, neurosurgery and otolaryngology clinical practices have begun employing AR, MR, and VR technologies. These XR technologies have been safely used to explore the operative field from various angles and visualize the neuroanatomy that is difficult to appreciate in the surgical field. This ability has improved the comprehensive sensory experience, particularly when using keyhole approaches to deeply situated targets (36, 37). AR not only facilitates accurate planning but also improves depth perception and reduces risks by superimposing segmented anatomical structures onto the operative field (29, 53, 62–70).

Given the dependence of AR on tracking strategies, imaging modalities, and the availability of anatomical registrations (16, 27, 71–73), Citardi et al. (74) recommended next-generation AR systems aim for a TRE of 0.6–1.5 mm for optimal performance. Birlo et al. (75) reviewed the use of optical see-through head-mounted displays in AR surgery and showed that they are mostly used in orthopedic surgery (28.6%), with a primary role in surgical guidance. The usefulness of optical see-through head-mounted displays was substantially affected by human variables. Clinical trials have revealed that the benefits of these devices are insufficient. They are not yet well-established in operating rooms. Their clinical utility would be ensured by a concentrated effort to resolve technical registration and perceptual variables in the laboratory as well as by a design that integrates human-factor considerations to address obvious clinical concerns. Despite challenges such as device bulkiness, limited battery life, and issues with legal implementation (76–78), AR continues to evolve, offering transformative potential in surgical simulations, training, and patient-specific interventions (38). These advancements highlight AR's promise in enhancing neurosurgical outcomes while emphasizing the need for refined technologies and streamlined integration.

MR is emerging as a transformative tool in skull base surgery and medical education. MR headsets combine spatial mapping, a hands-free interface, and MR vision to enable image-guided navigation. MR headsets use stereoscopy to project 3D models onto a transparent lens, allowing users to see holographic overlays without losing focus on their physical surroundings. This capability has been effectively utilized in surgical simulations, significantly enhancing neurosurgery and otolaryngology resident training (79–82). For example, a 2014 study by Hooten et al. demonstrated the educational value of MR through a ventriculostomy simulation in which virtual catheters were projected onto virtual ventricular models, offering an immersive learning experience (83). MR platforms are also revolutionizing intraoperative guidance, as Zeiger et al. showed in a study of endoscopic endonasal skull base surgeries using patient-specific 3D reconstructions (35), in which gross total resection was achieved in 56.3% of the cases. Furthermore, emerging technologies like Quicktome and Infinitome (Omniscient Neurotechnology) integrate Human Connectome Project data with MR for advanced surgical planning and training (84). These platforms, coupled with innovations such as Visualase (Medtronic), robotic auto-guide therapies, and stereoelectroencephalography-based implantation are advancing surgical precision and reducing morbidity (85). In a recent study by Bronowicki et al., who used the CarnaLife Holo system (MedApp S.A., Poland), the effects of MR on the lengths of the surgical procedure and hospitalization were evaluated in a surgically treated pediatric oncology cohort. The study compared the results of procedures with and without MR and found that hospitalization periods stayed within normal ranges and that surgical procedure length in the MR group was comparable to that for patients treated with traditional techniques. In a small number of cases performed with MR, a slight increase in procedure time was noted; however, this increase did not considerably lengthen the surgical procedure or hospitalization duration (86). With continuous improvements in computing power and machine learning, MR technologies are poised to further enhance skull base surgical care, improving both clinical outcomes and the educational landscape.

VR has emerged as a transformative tool in skull base surgery, offering detailed 3D models that enhance surgical planning, training, and intraoperative guidance. Introduced to the surgical discipline by Satava in 1993 (87), VR provides stereoscopic reconstructions of the surgical field, allowing surgeons to visualize complex anatomical relationships with depth and clarity (87–89). Techniques such as the 3D multifusion volumetric imaging, which integrates digital subtraction angiography, magnetic resonance imaging, and computed tomography, have demonstrated utility in preoperative planning for skull base tumors, improving approach selection and anticipation of intraoperative challenges (90). Recent studies, such as the 2021 study from Zawy Alsofy et al. (91), highlight VR's ability to optimize tumor-related anatomical visualization, refine surgical angles, and enhance safe and effective tumor resections. Additionally, Rosahl et al. combined VR with real-time infrared guidance in their virtual operating field to improve intraoperative anatomical navigation during skull base surgeries (33).

VR has also proven to be a valuable educational tool, enhancing surgical training through realistic anatomical simulations (92–97). Further studies, such as those using Stanford University's CardinalSim software for middle cranial skull base approaches, have demonstrated significant improvements in surgical proficiency, including reduced critical errors and faster procedure times (98). Innovations in photogrammetry have further advanced VR's educational applications, producing high-fidelity, photorealistic 3D models for neuroanatomical training (99, 100). For example, Corvino et al. created interactive models of the sellar region, enabling self-guided exploration and fostering a deeper understanding of the complex anatomy in the region (101). As these technologies evolve, VR is expected to become a cornerstone of skull base surgical education and practice, complementing cadaveric dissections and live surgeries while advancing precision and safety in patient care.

Surgeons could also use these systems to generate 3D images of anatomical structures in preoperative and intraoperative settings to provide crucial procedural guidance (102). Research has shown that these XR-based tools can enhance surgical outcomes and improve patient safety (102). Although it is evident that XR can play a critical role in skull base neurosurgery, it is important to fully understand the multiple ways in which it can be used. By identifying the strengths and gaps in this technology, engineers and clinicians can find novel ways to innovate upon these XR systems with the ultimate goal of improving patient outcomes and quality of life.

Findings from studies based on the cost-effectiveness of XR technologies in low- and middle-income countries (LMICs) showed positive and negative results. Stereoscopic XR proved to be helpful for new residents in obstetric emergency training according to a study by Bailey et al. (103). However, that study also pointed out that the use of XR in LMICs is constrained by its reliance on technology infrastructure. Although 83.8% of Pakistani healthcare professionals supported the educational benefits of XR, 70% of them cited technology infrastructure as a significant drawback, according to research by Khan et al. (104). Medical education in LMICs confronts two primary challenges according to a study by Li et al. (105). These challenges include a lack of resources and technology constraints. According to Mondal (106), India has conducted hardly any XR research (1.7% AR and 2.2% VR studies), although investigators also suggest more research be done. Developing cost-effective, open-source solutions along with policies promoting digital infrastructure and affordable tools can expand access, especially in the setting of LMICs (107, 108). Another potential strategy is to implement prototyping in these settings. XR prototyping can assist designers in producing low-cost iterative designs before manufacturers and investors decide to engage in research and development and manufacturing. XR prototyping is also more user-friendly than computer-based 3D modeling and displays 3D structures better than manual two-dimensional drawings (109).

VR is often associated with a steep learning curve, which limits a surgeon's ability to apply their preoperative knowledge in real-world patient settings (110). Long adaptive and training periods might be an additional hindrance to the smooth adoption of this technology into clinical practice. Overall, XR's incorporation into surgical planning and intraoperative guidance represents a paradigm shift in the way surgeons approach clinical practice. Traditional surgical training is successful, but it frequently follows a set format in which trainees move through predetermined learning phases at their own pace. A more flexible, individualized method would replace this rigidity with artificial intelligence-enhanced XR simulations, enabling surgeons to train at their own speed and concentrate on their most vulnerable areas. This customized training may greatly speed the development of skills and proficiency, allowing surgeons to become competent more quickly and confidently (111).

As XR technologies develop and become more integrated into healthcare procedures, it will be important to recognize and treat the possible long-term clinical effects, both positive and negative. It is important to carefully plan and execute XR solutions to optimize advantages, reduce risks, and guarantee equitable access and ethical use. Standardized outcome reporting and continuous monitoring are necessary for future studies in order to completely understand and reduce the possible long-term impacts of XR on clinicians' health and well-being. Future developments should focus on cost-effective, user-friendly designs, leveraging artificial intelligence and photogrammetry to optimize 3D modeling and XR systems. Collaborative efforts and prospective research are crucial for refining these technologies, integrating them seamlessly into surgical workflows, and improving surgical outcomes while ensuring patient safety. Table 6 provides a brief summary of the role of XR in skull base surgery.

**Table 6:** Summary of XR use in skull base surgery.

Parameter	AR	VR	MR
Required equipment	Microscope overlays, projectors, half-silvered mirrors, mobile devices, head-mounted displays, external tracking hardware, optical tracking cameras	Simulator platform software, haptic device, segmentation software	Head-mounted display, segmentation software, optical tracking cameras, transparent lens
Cost	$120–$250 per min	$100,000–$110,000	$179,000–$300,000
Time to implement	Registration of the skull phantom targeting the exterior of the skull: 1–5 min; annotation of the virtual pathways, registration, and the procedure requiring navigation of the needle: 3–5 min	Model construction takes 1–2 h depending on the number and complexity of the segmented structures	CT acquisition: 3–4 min for each scan; automated volume rendering and scan fusion: 3–5 min; model construction: 10 min–1 h
Future indications	AR-VR integration: a paradigm change could result from using VR and AR templates based on the anatomy of a specific patient, in which the surgeon performs the procedure by repeating the previously practiced plan; enhanced preoperative planning: critical anatomic landmarks can be identified that are expected to facilitate dissections, and annotation of these points, measurements, and trajectories can then be presented to surgeons in an AR system in addition to displaying virtual models of patient-specific anatomy	Simultaneous navigation and dissection: including incorporation of dedicated nasal instruments and the implementation of dual haptics that will allow for simultaneous endoscopic navigation and dissection	Automatic registration: by allowing the hologram to be automatically superimposed onto the anatomy of interest and to the skin marker in the planning phase

AR, augmented reality; CT, computed tomography; MR, mixed reality; VR, virtual reality; XR, extended reality.

### 4.1 Limitations

Despite their transformative potential, XR applications in skull base surgery face several limitations. “Crowding of objects” in digital overlays can overwhelm surgeons and reality algorithms, necessitating interfaces that prioritize critical information to reduce cognitive overload (112). Additionally, head-mounted devices remain cumbersome, causing physical fatigue during extended procedures, which could potentially reduce accuracy and negatively affect outcomes; lighter ergonomic designs and alternative display technologies, such as integrated operating room systems, are needed (113, 114). Oculomotor abnormalities, nausea, disorientation, and discomfort were among the common negative effects of virtual reality shown by a recent systematic study by Cossio et al. (115). Furthermore, current magnetic resonance scanning resolution limits accurate anatomical segmentation, although advancements in artificial intelligence and high-resolution imaging could address these challenges (116). Ensuring precise overlays demands extensive hardware-software integration, with ongoing efforts required to enhance system reliability, minimize latency, and improve calibration (117, 118). Data security, data management, and the potential for attention bias further underscore the importance of tailored interfaces and robust encryption methods (110). The cost associated with establishing these technologies also poses a significant challenge, especially in hospitals in LMICs.

## 5 Conclusion

The integration of XR technologies into skull base surgery has the potential to change surgical planning, training, teaching, and intraoperative guidance. These technologies enhance visualization of complex anatomical structures, improve surgical precision, and provide immersive educational tools that complement traditional training methods. Despite limitations such as poor device ergonomics, resolution constraints, and the need for robust system integration, ongoing advancements in artificial intelligence, photogrammetry, and imaging modalities promise to address these challenges. By fostering interdisciplinary collaboration, developing user-friendly interfaces, and ensuring rigorous evaluation of clinical outcomes, XR systems can be seamlessly integrated into skull base surgical workflows.

## Data Availability

The original contributions presented in the study are included in the article, and further inquiries can be directed to the corresponding author.
